# The chaperone protein HSP47: a platelet collagen binding protein that contributes to thrombosis and hemostasis

**DOI:** 10.1111/jth.13998

**Published:** 2018-04-15

**Authors:** P. Sasikumar, K. S. AlOuda, W. J. Kaiser, L. M. Holbrook, N. Kriek, A. J. Unsworth, A. P. Bye, T. Sage, R. Ushioda, K. Nagata, R. W. Farndale, J. M. Gibbins

**Affiliations:** ^1^ Institute for Cardiovascular and Metabolic Research School of Biological Sciences University of Reading Reading UK; ^2^ Laboratory of Molecular and Cellular Biology Faculty of Life Sciences Kyoto Sangyo University Kyoto Japan; ^3^ Department of Biochemistry University of Cambridge Cambridge UK

**Keywords:** chaperone, hemostasis, HSP47, platelets, thrombosis

## Abstract

**Essentials**

- Heat shock protein 47 (HSP47), a collagen specific chaperone is present on the platelet surface.
- Collagen mediated platelet function was reduced following blockade or deletion of HSP47.
- GPVI receptor regulated signalling was reduced in HSP47 deficient platelets.
- Platelet HSP47 tethers to exposed collagen thus modulating thrombosis and hemostasis.

**Summary:**

**Objective**

Heat shock protein 47 (HSP47) is an intracellular chaperone protein that is vital for collagen biosynthesis in collagen secreting cells. This protein has also been shown to be present on the surface of platelets. Given the importance of collagen and its interactions with platelets in triggering hemostasis and thrombosis, in this study we sought to characterize the role of HSP47 in these cells.

**Methods and Results**

The deletion of HSP47 in mouse platelets or its inhibition in human platelets reduced their function in response to collagen and the GPVI agonist (CRP‐XL), but responses to thrombin were unaltered. In the absence of functional HSP47, the interaction of collagen with platelets was reduced, and this was associated with reduced GPVI‐collagen binding, signalling and platelet activation. Thrombus formation on collagen, under arterial flow conditions, was also decreased following the inhibition or deletion of HSP47, in the presence or absence of eptifibatide, consistent with a role for HSP47 in enhancing platelet adhesion to collagen. Platelet adhesion under flow to von Willebrand factor was unaltered following HSP47 inhibition. Laser‐induced thrombosis in cremaster muscle arterioles was reduced and bleeding time was prolonged in HSP47‐deficient mice or following inhibition of HSP47.

**Conclusions**

Our study demonstrates the presence of HSP47 on the platelet surface, where it interacts with collagen, stabilizes platelet adhesion and increases collagen‐mediated signalling and therefore thrombus formation and hemostasis.

## Introduction

Heat shock protein 47 (HSP47) is a 400 amino acid residue glycoprotein that is predominantly localized within the secretory system of collagen‐producing cells 1. Here it functions as a chaperone for newly synthesized procollagens, stabilizing the triple helical structures that procollagen trimers adopt 2, 3. Transgenic mice that lack both alleles of the HSP47 gene (serpinH1) die *in utero* with the appearance of abnormally deposited collagen basement membranes. Because abnormal collagen synthesis underpins a host of fibrotic diseases, such as liver sclerosis or pulmonary fibrosis, HSP47 is of interest as a potential therapeutic target for such disorders 4.

Despite its well‐characterized role as an intracellular collagen chaperone, HSP47 was originally discovered on the surface of mouse embryo parietal endoderm cells 5. On these cells it was suspected to function as a receptor for extracellular collagens, because it binds to native collagen type IV and gelatin *in vitro*. Human oral squamous carcinoma cells and chondrocytic cell lines were also reported to expose HSP47 on their surfaces 6, 7. All three cell types synthesize collagen, so it is unclear whether the role of HSP47 on the plasma membrane was secondary to its chaperone activity.

We have previously reported that the peripheral membrane fraction isolated from human platelets contained HSP47 8. Platelets are anucleate, non‐collagen producing cells that lack the classical ER system. However, they interact with exposed collagen at sites of vessel wall injury to form thrombi and plug the wound, thus preventing blood loss. Our observation that HSP47 is exposed on the platelet surface led to the hypothesis that HSP47 may itself influence the interaction of platelets with collagen following vessel injury.

In this study, we report that platelet HSP47 strengthens platelet interactions with collagen in the formation of thrombi and hemostasis, revealing a fundamental new mechanism for chaperone protein function in the extracellular regulation of cellular function.

## Materials and methods

### Ethics statement

Human blood collection was performed with approval from the University of Reading Local Ethics Review Panel. Experiments involving the use of animals (mice) were performed in accordance with a licence from the UK Home Office.

### Immunofluorescence microscopy

Primary mouse megakaryocytes were isolated from C57Bl/6 mice femur bone marrow as described previously 9. Fixed, non‐permeabilized primary megakaryocytes were stained with monoclonal rabbit anti‐HSP47 (EPR4217, Abcam, Cambridge, UK) or control IgG, then immunodetected with Alexa fluor 488 labelled anti‐rabbit antibodies and co‐stained with Alexa 647 labeled GPIb. The images were visualized by epi‐fluorescence microscopy (Leica Zeiss, Cambridge, UK, 100× oil immersion).

### Cell surface biotinylation of HSP47

Cell surface biotinylation was performed as described previously 9. The presence of HSP47 was analyzed by immunoblotting and detected with anti‐HSP47 (mouse anti‐HSP47, clone M16.10A1, Stressgen Biotechnologies, Victoria, BC, Canada; 1:500), and the immunoblots were re‐probed using streptavidin‐HRP to reveal total cell‐surface biotinylation and glyceraldehyde‐3‐phosphate dehydrogenase (GAPDH) antibody (Abcam) as a negative control for cytosolic proteins.

### Collagen sepharose affinity chromatography

A total of 100 μg mL^−1^ collagen type I or bovine serum albumin (BSA) (protease free) was coupled to cyanogen bromide‐activated sepharose 4B (Sigma, Dorset, UK) as per the manufacturer's instructions. Washed platelets (4 × 10^8^ cells) were incubated with either small molecule inhibitor of HSP47 (SMIH, compound IV, was identified from a large‐scale screen of compounds that interfered with HSP47 interaction with collagen and prevents its fibrillogenesis 10; Maybridge, RH00007SC, 20 μm, Thermo fisher scientific, Leicestershire, UK), vehicle (0.1% dimethylsulfoxide [DMSO]) or inhibitory rabbit polyclonal anti‐HSP47 (anti‐HSP47; the antibody was raised against synthetic peptide corresponding to the collagen binding site [amino acid residues 406–417] of human HSP47 [lyophilized‐azide free]; Life Span Biosciences, Nottingham, UK), control rabbit IgG for 2 min at 37 °C and lysed with 2% (v/v) Nonidet P40 buffer with protease inhibitors. Either collagen or BSA‐sepharose were added and rotated for 1 h at 4 °C. Following centrifugation, pellets were re‐suspended in reducing Laemmli buffer. Protein samples were assessed by immunoblot analysis using mouse HSP47 and GPVI antibodies (anti‐human GPVI [AF3627]). Cy2 anti‐sheep IgG and Cy5 donkey anti‐mouse IgG (1:1000) were used for the detection of protein bands using Typhoon Trio Fluorescence Imager (GE Healthcare, Buckinghamshire, UK).

### Preparation of washed human platelets, aggregation studies, ATP secretion and calcium measurement

Human blood was obtained from healthy, drug‐free volunteers into 4% (w/v) sodium citrate and washed platelets were isolated as described previously 11. Using an optical aggregometer, washed human platelets (4 × 10^8^ platelets mL^−1^) were incubated with rabbit polyclonal function blocking anti‐HSP47 (anti‐HSP47) or the rabbit IgG at 37 °C for 2 min, followed by stimulation with collagen (collagen type I fibres) (Nycomed, Munich, Germany), CRP‐XL and thrombin whilst stirring, and aggregation traces were recorded.

Calcium measurement was performed as described previously 11. Fura‐2 loaded platelets were incubated with anti‐HSP47 or control IgG for 2 min at 37 °C prior to addition of agonists. ATP secretion assays were performed using luciferin‐luciferase luminescence substrate as described previously 11. Platelets (4 × 10^8^cells mL^−1^) were pre‐incubated with luciferase prior to the addition of anti‐HSP47 or control IgG.

### Generation of HSP47 conditional knockout mice

Mice homozygous for the HSP47 floxed allele (loxP sites flanking exon 6) on a C57BL/6 background were generated as previously reported 12. Mice lacking HSP47 in platelets (Pf4‐Cre; HSP47^*flox/flox*^) were generated by crossing HSP47 floxed mice with Pf4‐Cre mice (Jackson Laboratories, Bar Harbor, ME, USA) expressing Cre recombinase under control of the Pf4 promoter 13.

### Preparation of washed mouse platelets, immunoblot analysis, aggregation studies, receptor expression studies and signaling studies

Mouse blood was collected by cardiac puncture and washed platelets prepared as described previously 11. Mouse platelet protein lysates were assessed by immunoblot analysis using rabbit anti‐HSP47 antibody. Blots were re‐probed with anti‐actin (C‐11, Santa Cruz, Heidelburg, Germany) to control for protein loading levels. For aggregation studies, washed mouse platelets (2 × 10^8^ cells mL^−1^) were stimulated with collagen and aggregation traces recorded. Whole mouse blood flow cytometry studies were performed 14 to measure receptor expression levels on platelets using fluorescein isothiocyanate (FITC)‐conjugated antibodies for GPVI, integrin α_IIb_β_3_, integrin α_2_β_1_ and GPIbα (Emfret Analytics, Eibelstadt, Germany). Signaling studies experiments were performed as described previously 15. Mouse platelets (4 × 10^8^ cells mL^−1^, HSP47^*flox/flox*^ or Pf4‐Cre; HSP47^*flox/flox*^) were either untreated or stimulated with 1 μg mL^−1^ CRP‐XL for 90 s at 37 °C, after which reducing SDS‐PAGE sample buffer was added to stop the reaction. Immunoblotting and band intensity analysis was performed as described previously 15.

### *In vitro* thrombus formation

Vena8 Biochips (Dublin, Ireland) were coated with collagen (400 μg mL^−1^) or von Willebrand factor (VWF) (400 μg mL^−1^) in Tyrodes‐HEPES buffer overnight at 4 °C then blocked for 1 h with 1% (w/v) BSA. DiOC_6_ (0.87 μm DiOC_6_, 0.05% ethanol) labeled citrated mouse blood was perfused over collagen‐coated surface at a shear rate of 1000 s^−1^ for 10 min. Thrombi were visualized using a Nikon eclipse (TE2000‐U) inverted microscope (Nikon Instruments, Surrey, UK) (N PLANL 10 x objective) and analyzed using Slidebook 5.5.

For experiments using SMIH and inhibitory anti‐HSP47, fluorescently labeled human blood was incubated with either SMIH or vehicle control (0.1% [v/v] DMSO) or inhibitory anti‐HSP47 or control IgG for 2 min at 37 °C. Blood was then perfused through glass capillary slides (Camlab, Cambridge, UK) coated with collagen or VWF (400 μg mL^−1^) at a shear rate of 1000 s^−1^ for 8 min. Thrombi were visualized using a Leica DMIRE2 inverted confocal microscope (20x/0.4) and analyzed using TCS SP2 software (Leica, Mayfair, London, UK).

To study single platelet adhesion to collagen, blood was pre‐incubated for 2 min with eptifibatide (4 μm eptifibatide; Fluorochem, Derbyshire, UK) prior to addition of HSP47 inhibitor (20 μm SMIH) or vehicle control, or rabbit polyclonal anti‐HSP47 or control IgG was perfused over collagen‐coated glass capillary slides for 8 min. Each slide was flushed with TBS/1% (v/v) NP40 buffer and eluted. Platelet adhesion was measured either from fluorescence intensity (Fiji, Image J, https://imagej.nih.gov/ij/) or by protein estimation using BCA protein assay (Pierce, Paisley, UK).

### Laser injury of mouse microvessels and tail bleeding assay

*In vivo* analysis of thrombosis using a laser injury model and tail bleeding assays were performed as described previously 11, 16, 17. For experiments with HSP47 inhibitor, SMIH (achieved a concentration in the blood of approximately 20 μm) or vehicle control (0.1% [v/v] DMSO) was infused into the mouse (C57Bl/6) circulation 5 min prior to laser injury or removal of tail tip. For the thrombosis assay, GPIβ subunit antibody was used for *in vivo* platelet labelling (X488, Emfret Analytics). The same procedure was used when thrombosis and tail bleeding assays were performed for Pf4‐Cre; HSP47^*flox/flox*^ and HSP47^*flox/flox*^.

### Statistics

For two‐group comparisons, Student's *t*‐test was used; *P* values of < 0.05 were considered statistically significant (*n* = 3 or more). Normalized data were subjected to statistical analysis prior to normalization. Two‐way anova was used for *in vitro* thrombus formation assay (GraphPad Software, San Diego, CA, USA). The non‐parametric Mann–Whitney test was used to analyze non‐normally‐distributed data (tail bleeding assay).

## Results

### Platelet‐derived HSP47 is expressed constitutively on the platelet surface and has megakaryocytic origins

**Figure 1:**
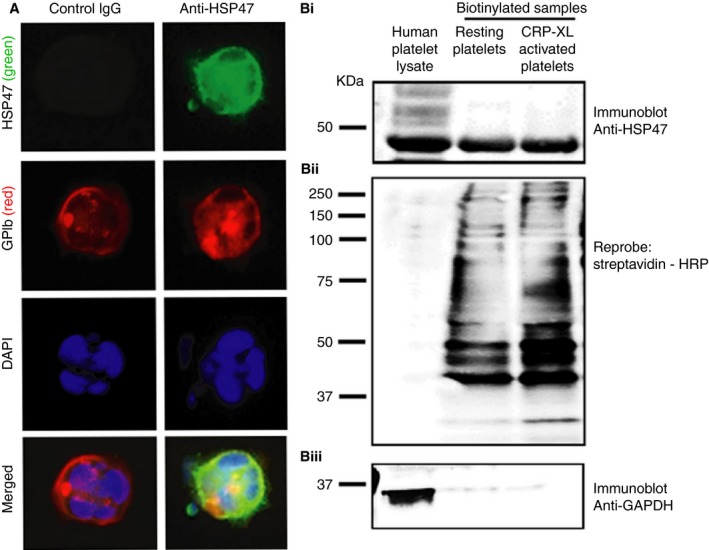
HSP47 is present on the surface of platelet progenitor primary megakaryocytes and platelets. The presence of HSP47 on the surface of primary megakaryocytes isolated from mouse bone marrow was demonstrated using epi‐fluorescence microscopy under non‐permeabilizing conditions using rabbit monoclonal anti‐HSP47 antibody. (A) Representative images for HSP47 (green), GPIb (red) and DAPI (blue). (B) The presence of HSP47 at the human platelet surface was evaluated by means of a biotin‐based labelling approach. Resting (Tyrodes‐HEPES buffer treated) and stimulated (2 μg mL ^−1^ CRP‐XL) platelet cell‐surface proteins (from 1 × 10^9^ cells) were biotinylated using EZ link sulfo LC‐NHS biotin and purified from platelet lysates by NeutrAvidin affinity chromatography. (Bi) Biotinylated platelet surface proteins were probed using anti‐HSP47 antibody. (Bii) Immunoblots were re‐probed with streptavidin‐HRP conjugate to reveal total biotinylation. (Biii) Immunoblots were also re‐probed for glyceraldehyde‐3‐phosphate dehydrogenase (GAPDH), a cytosolic protein. Data is representative of three separate experiments. [Color figure can be viewed at wileyonlinelibrary.com]

Previous proteomics studies demonstrated the presence of HSP47 in the peripheral membrane fraction of human platelets 8, 18. Further flow cytometric analysis of washed platelets (Figure S1Ai, Aii, Aiii, Aiv) and whole blood analysis (data not shown) confirmed that resting human platelets, which do not possess P‐selectin on the cell surface, display detectable levels of HSP47 immunoreactivity on their surface. Being anucleate, platelet protein content is derived almost exclusively from the progenitor megakaryocyte during megakaryopoiesis. Consistent with this, primary mouse megakaryocytes exhibited detectable levels of HSP47 immunoreactivity on their surface membrane (Fig. 1A). Further to this, to confidently establish the presence of HSP47 on the outer leaflet of the platelet plasma membrane we carried out cell surface biotinylation and purification of biotinylated human platelet proteins. Immunoblot analysis of isolated biotinylated surface proteins of resting and CRP‐XL (crosslinked collagen‐related peptide) stimulated human platelets revealed HSP47 to be constitutively present on the platelet plasma membrane (Fig. 1Bi). Figure 1(Bii) confirms that a range of membrane proteins were biotinylated and revealed differential biotin labeling between resting and stimulated human platelets, consistent with the known upregulation of proteins on the platelet surface upon activation. However, although by flow cytometry an increase in cell surface HSP47 can be detected 8, an increase in biotinylated proteins from CRP‐XL‐activated human platelets was not observed. This could be a result of the biotinylation reagent gaining access to the HSP47 in the open canicular system in addition to the platelet surface. HSP47 in the open canicular system may represent the origins of increased surface levels upon activation. The cytosolic protein GAPDH was used as a control to confirm that biotinylation of proteins was confined to the platelet surface (Figure 1Biii).

### Platelet‐derived HSP47 associates with collagen fibrils and modulates GPVI adhesion to collagen

**Figure 2:**
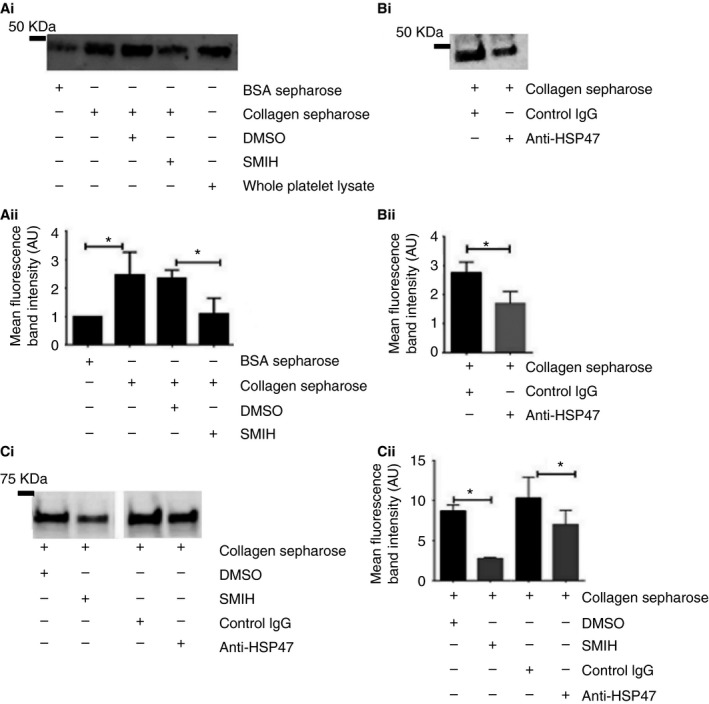
Platelet HSP47 binds to immobilized collagen fibrils and can modulate GPVI–collagen interaction. Isolated washed human platelets were lysed and incubated with either collagen sepharose or bovine serum albumin BSA‐sepharose. For experiments with small molecule inhibitor of HSP47 (SMIH), platelets were preincubated with 20 μm SMIH or vehicle control dimethylsulfoxide (DMSO) (0.1%) prior to the addition of collagen sepharose. (Ai) The bound proteins were visualized using immunoblot analysis. (Aii) Data represent mean ± SD (*n* = 3). Student's *t*‐test, *P < 0.05. The basal level band intensity obtained from bovine serum albumin (BSA) sepharose was taken as 100% and data normalized to this value. For experiments with inhibitory anti‐HSP47, platelets were preincubated with control IgG (10 μg mL ^−1^) or anti HSP47 (10 μg mL ^−1^) prior to the addition of collagen sepharose. (Bi) The bound HSP47 was visualized using immunoblot analysis. (Bii) Data represent mean ± SD (*n* = 4). Student's *t*‐test, *P < 0.05. For experiments to evaluate bound GPVI, platelets were preincubated with control IgG (10 μg mL ^−1^) or anti‐HSP47 (10 μg mL ^−1^), SMIH (20 μm) or vehicle control (0.1% DMSO) prior to the addition of collagen sepharose. (Ci) Bound GPVI was visualized by immunoblot analysis, (Cii) Cumulative data represent mean ± SD (*n* = 4). Student's *t*‐test, *P < 0.05.

Given the importance of collagen in the initiation of platelet function, we asked whether platelet HSP47 interacts with collagen. Platelet HSP47–collagen interaction was investigated by means of collagen sepharose affinity chromatography. Immunoblot analysis revealed that platelet HSP47 present in the cell lysates bound to collagen sepharose beads with only limited interaction with BSA‐coupled beads. The level of HSP47 binding was reduced to background binding levels (equivalent to BSA–sepharose binding) in the presence of the small molecule inhibitor of HSP47 (SMIH; Fig. 2Ai, Aii). Similarly, we studied platelet HSP47–collagen sepharose interaction in the presence of inhibitory anti‐HSP47. Immunoblot analysis showed that HSP47 protein bound to collagen sepharose was reduced (Fig. 2Bi, Bii). To determine whether platelet HSP47 influences platelet interactions with collagen, we investigated whether GPVI binding to collagen is regulated in the presence of HSP47 inhibitors. Immunoblot analysis revealed that GPVI bound to collagen sepharose beads is reduced in the presence of both HSP47 inhibitors (SMIH and inhibitory anti‐HSP47; Fig. 2Ci, Cii).

Flow cytometry analysis was employed to determine whether HSP47 influences the ability of platelets to bind to collagen. Binding of FITC‐labeled collagen to human platelets is reduced in the presence of small molecule inhibitor (20 μm) or inhibitory HSP47 antibody (10 μg mL^−1^). There was no additional reduction in FITC‐labeled collagen binding to platelets in the presence of both SMIH and inhibitory anti‐HSP47 (Figure S1B). Furthermore, on resting platelets the surface expression of the receptors GPVI, integrin α_2_, integrin β_3_ and GPIb in the presence of SMIH or inhibitory anti‐HSP47 or in combination was unaltered (Figure S1C, D, E, F).

### Targeting surface‐exposed HSP47 with antibodies attenuates collagen‐induced human platelet aggregation

**Figure 3:**
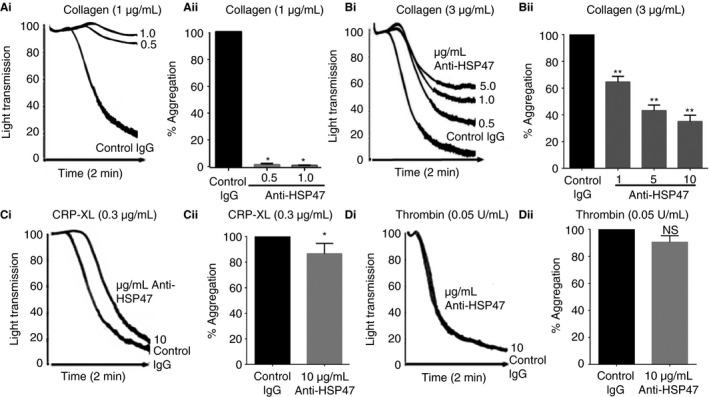
Isolated, washed human platelets were stimulated with type I collagen fibrils in the presence of function blocking rabbit polyclonal Anti‐HSP47 or rabbit IgG (Control IgG). Aggregation responses were recorded following stimulation with 1 μg/mL collagen (Ai), 3 μg/mL (Bi), 0.3 μg/mL CRP‐XL (Ci) or 0.05U/mL thrombin (Di). Data (Aii, Bii, Cii, Dii) represent mean ± S.D (*n*=3) one way ANOVA (**P < 0.01 and *P < 0.05) for collagen data and Student's *t*‐test (*P < 0.05) for CRP‐XL or thrombin. Aggregation obtained with control IgG was taken as 100% and was normalised to this value.

We have demonstrated previously that the HSP47 inhibitor SMIH (previously referred to as inhibitor of HSP47 [IOH] 8) reduced platelet aggregation, particularly in response to collagen. Because we cannot exclude that the small molecule inhibitor could gain access to intracellular HSP47, here surface‐expressed HSP47 was targeted using a function blocking rabbit anti‐HSP47 polyclonal antibody (Anti‐HSP47). Full platelet aggregation, stimulated with 1 μg mL^−1^ collagen type I fibrils, was prevented completely with 1 μg mL^−1^ anti‐HSP47 (Fig. 3Ai, Aii). Higher concentrations of collagen (3 μg mL^−1^) partially overcame the inhibition (Fig. 3Bi, Bii). Low levels of inhibition were observed when platelets were incubated with 10 μg mL^−1^ anti‐HSP47 and then stimulated with 0.3 μg mL^−1^ CRP‐XL (Fig. 3Ci, Cii). Thrombin (0.05 U mL^−1^)‐stimulated aggregation was unaffected in the presence of inhibitory antibody (Fig. 3Di, Dii). Aggregation upon stimulation with convulxin, which potently stimulates GPVI, was not reduced in the presence of anti‐HSP47 (Figure S2A, Aii), indicating that HSP47 functions exclusively through a collagen (or collagen like, in the case of CRP‐XL) mediated response. To explore the specificity of action for HSP47 in platelets, we investigated the effect of HSP47 on aggregation responses to a range of concentrations of collagen, CRP‐XL or thrombin using a 96‐well plate‐based aggregation assay. At a concentration of collagen (3 μg mL^−1^) and CRP‐XL (0.3 μg mL^−1^) that elicited comparable kinetics and extent of aggregation, a reduction in platelet aggregation in the presence of anti‐HSP47 (10 μg mL^−1^ anti‐HSP47, the highest concentration of inhibitory antibody, was selected to compare with inhibition observed with light transmission aggregometry) was observed (Figure S2A, B). In contrast, no reduction in aggregation was observed at any concentration of thrombin tested (Figure S2C).

### Platelet HSP47 modulates calcium mobilization and dense granule secretion

**Figure 4:**
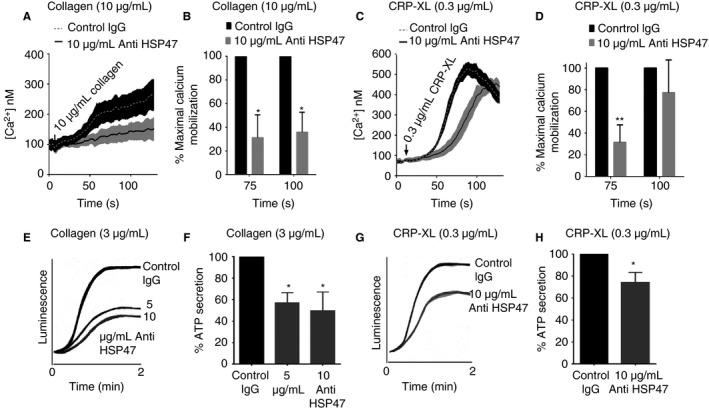
Inhibition of HSP47 function reduces calcium mobilization and ATP secretion. Calcium mobilization was measured by spectrofluorimetry in Fura‐2 AM loaded platelets at excitation at 340/380 nm and emission at 510 nm. Platelets preincubated with anti‐HSP47 (10 μg mL ^−1^) or control IgG (10 μg mL ^−1^) were stimulated with 10 μg mL ^−1^ of collagen (A, B) or 0.3 μg mL ^−1^ of CRP‐XL (0.3 μg mL ^−1^) (C, D). The traces shown are representative of four separate experiments. The maximal calcium level obtained with control IgG was taken as 100% and data normalized to this value. The level of ATP secretion was measured using lumino‐aggregometry following stimulation with 3 μg mL ^−1^ of collagen (E, F) and 0.3 μg mL ^−1^ of CRP‐XL (G, H). Traces shown here are representative of three separate experiments. Data represent mean ± SD (*n* = 3; Student's *t*‐test, *P < 0.05). ATP secretion obtained with control IgG was taken as 100% and data were normalized to this value.

Ca^2+^ mobilization plays a pivotal role in various aspects of platelet functions, such as shape change and degranulation; hence, we investigated the role of HSP47 in this process. Ca^2+^ levels were measured following collagen stimulation for 75 and 100 s, and were reduced significantly by 69% and 70%, respectively, in comparison with control IgG (Fig. 4A, B). A higher concentration of collagen (10 μg mL^−1^) was required to stimulate detectable Ca^2+^ levels under non‐stirring conditions, consistent with the previous studies 18. Similarly, when platelets were stimulated with CRP‐XL (0.3 μg mL^−1^) in the presence of anti‐HSP47 a significant reduction of ~68% at 75 s was observed; however, at a later time‐point of 100 s the reduction was partially overcome (Fig. 4C, D). It is interesting to note that although collagen‐mediated Ca^2+^ mobilization was inhibited, CRP‐XL‐mediated responses were only delayed.

Because calcium mobilization is known to modulate dense granule secretion, ATP secretion was assessed in the presence of inhibitory anti‐HSP47. ATP secretion was reduced by approximately 50% and 26% in the presence of anti‐HSP47 (10 μg mL^−1^) compared with control IgG in platelets activated with collagen (3 μg mL^−1^) or CRP‐XL (0.3 μg mL^−1^), respectively (Fig. 4E–H).

### Characterization of Pf4‐Cre; HSP47^flox/flox^ mouse platelets

**Figure 5:**
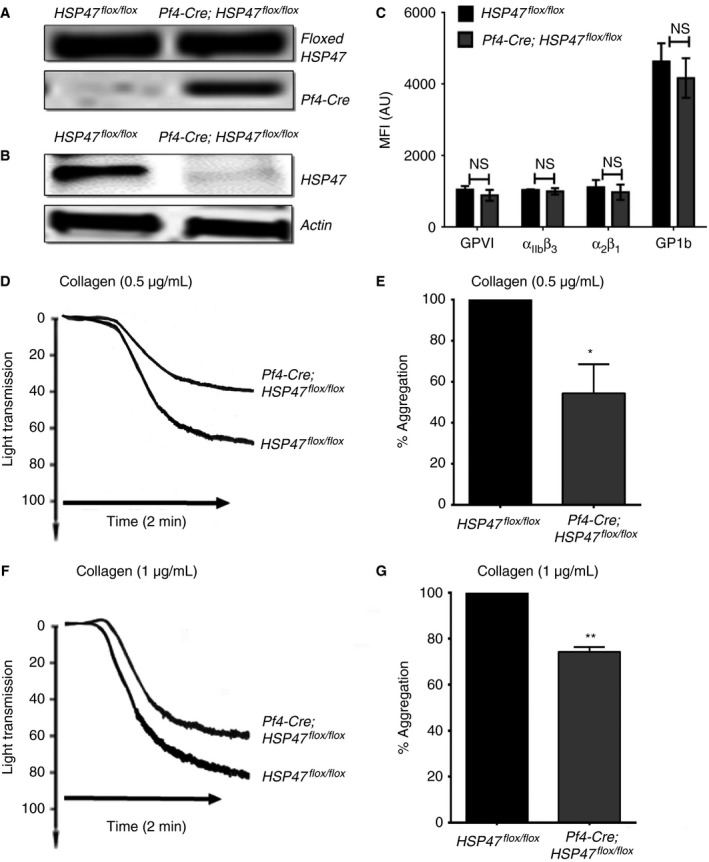
HSP47‐deficient platelets display diminished response to collagen or CRP‐XL. Mice lacking platelet HSP47(Pf4‐Cre; HSP47^*flox/flox*^) were generated using the recombination strategy mediated by expression of Cre‐recombinase in megakaryocytes. (A) PCR analysis of genomic DNA from Pf4‐Cre; HSP47^*flox/flox*^ and HSP47^*flox/flox*^ ear tissue samples. (B) Immunoblot analysis of mouse platelet lysates at concentration 4 × 10^8^ cells/ml to detect HSP47 protein. (C) The expression levels of αIIbβ3, GPVI, α2β1 and GPIbα analyzed by flow cytometry using citrated mouse blood Pf4‐Cre; HSP47^*flox/flox*^ or HSP47^*flox/flox*^ mouse blood. Data represent mean ± SD (*n* = 3). *P*‐values calculated by Student's *t*‐test (*P* > 0.05). Washed platelets from HSP47^*flox/flox*^ and Pf4‐Cre; HSP47^*flox/flox*^ mice were stimulated with 0.5 μg mL ^−1^ collagen (D, E) or 1.0 μg mL ^−1^ collagen (F, G). Cumulative data are presented as mean ± SD (*n *= 4). 100% aggregation is defined as the level of aggregation obtained with HSP47^*flox/flox*^ (control) (Student's *t*‐test, **P < 0.01 and *P < 0.05).

Mice lacking platelet HSP47 were generated using the recombination strategy mediated by expression of Cre recombinase in megakaryocytes. Generation of HSP47^*flox/flox*^ (control) and Pf4‐Cre; HSP47^*flox/flox*^ (mice lacking platelet HSP47) mice were confirmed by PCR analysis of genomic DNA isolated from ear tissue samples (Fig. 5A). The absence of platelet HSP47 in Pf4‐Cre; HSP47^*flox/flox*^ mice was demonstrated by immunoblot analysis using rabbit anti‐HSP47 (Fig. 5B). A full uncropped representative blot including HSP47^*flox/flox*^, Pf4‐Cre; HSP47^*flox/flox,*^ human and C57BL/6 platelets probed with anti‐HSP47 is shown in Figure S3(A). Expression levels of platelet receptors integrins α_IIb_β_3_, α_2_β_1_, GPVI and GPIb (Fig. 5C) on HSP47^*flox/flox*^ and Pf4‐Cre; HSP47^*flox/flox*^ platelets were found to be comparable. Platelet counts were also analyzed and found to be unaffected by HSP47 deletion (Figure S3B).

### Mouse platelets lacking HSP47 exhibit reduced platelet activation

Platelet aggregation assays were performed to investigate the effect of deletion of HSP47 on platelet function. Aggregation of washed platelets lacking HSP47 (Pf4‐Cre; HSP47^*flox/flox*^) was reduced by ~40% at a concentration of 0.5 μg mL^−1^ collagen (Fig. 5D, E) compared with control (HSP47^*flox/flox*^) mouse platelets. At a higher concentration of collagen (1 μg mL^−1^), the reduction was maintained but was less pronounced at ~30% (Fig. 5F, G), consistent with the ability of high agonist concentrations to overcome inhibition of HSP47 in human platelets (Fig. 3G, H). Following stimulation with CRP‐XL, mouse platelets lacking HSP47 showed reduced aggregation and fibrinogen binding compared with control mice (Figure S4Ai, Aii, B). However, no reduction in fibrinogen binding was observed upon thrombin stimulation of platelets lacking HSP47 in comparison with control platelets (Figure S4B).

To establish whether the effects of SMIH or anti‐HSP47 could be attributed specifically to actions on platelet HSP47, we investigated their actions on control and HSP47‐deficient platelets. Prior to this, the ability of HSP47 inhibitors to modulate murine (C57BL/6) platelet function was tested and they were found to reduce the mouse platelet aggregation (Figure S5Ai, Aii). Neither the small molecule inhibitor of HSP47 nor the inhibitory anti‐HSP47 antibodies displayed any inhibitory effect on HSP47‐deficient platelets, confirming the specificity of SMIH or antibody‐mediated effects (Figure S5Bi, Bii, Ci, Cii).

### HSP47 plays a significant role in thrombus formation *in vitro*

**Figure 6:**
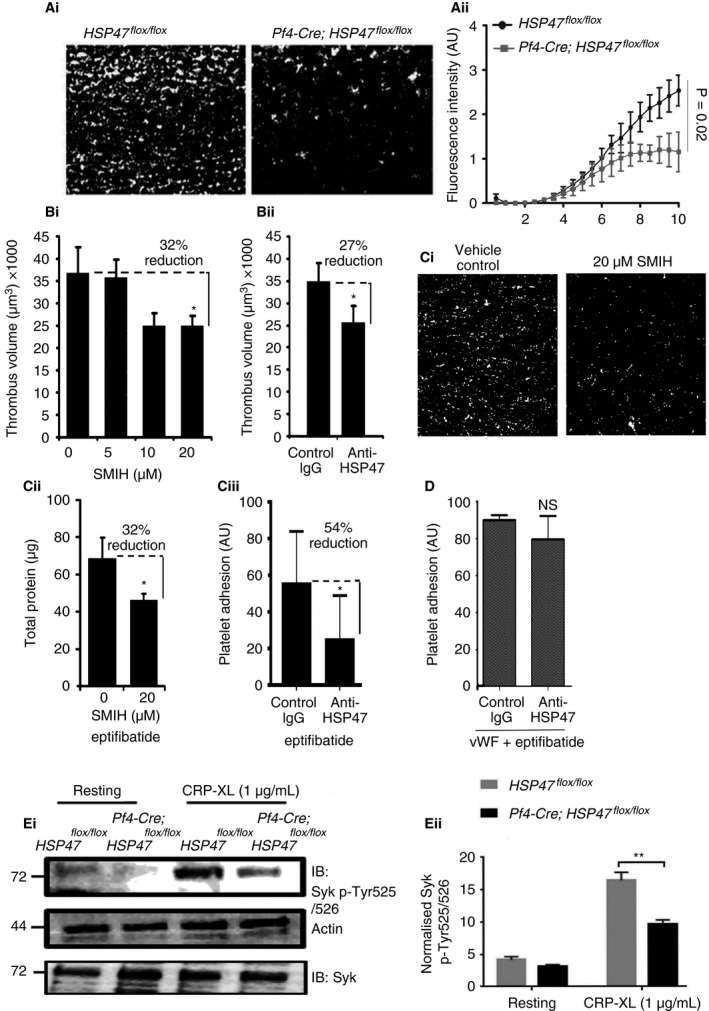
HSP47 supports the initial adhesion of platelets to collagen, thus enhancing GPVI signaling. Blood drawn from HSP47^*flox/flox*^ and Pf4‐Cre; HSP47^*flox/flox*^ mice labeled with DiOC6 was perfused over collagen‐coated surfaces at a wall shear rate of 1000 s^−1^ for 10 mins. (Ai) Representative images are shown. Captured images of adherent thrombi were analyzed by calculating the sum intensity of fluorescence as a measure of thrombus formation. (Aii) Surface coverage at 10 min is presented as mean ± SD (*n *= 3); *P* = 0.02 by two‐way anova. (Bi) Anticoagulated human blood without eptifibatide was labeled with DiOC6 incubated with small molecule inhibitor of HSP47 (SMIH) or controls (dimethylsulfoxide [DMSO]) at (1000 s^‐1^) for 4 mins. Thrombus volume was measured by confocal microscopy following incubation of blood with 5 μm, 10 μm and 20 μm SMIH (mean ± SD,* n* = 6, *P < 0.05). (Bii) Thrombus volume was measured following incubation of blood with function blocking anti‐HSP47 or control IgG (mean ± SD,* n* = 6, *P < 0.05). (Ci) Representative fields of thrombi formed under similar conditions with SMIH (20 μm) or vehicle control treatment in the presence of eptifibatide (4 μm). (Cii) The levels of platelet adhesion to collagen were measured by analysis of concentration of protein eluted from flow cells after perfusion (mean ± SD,* n* = 5, *P < 0.05). (Ciii) Platelet adhesion was measured after incubation of human blood with anti‐HSP47 or control IgG in the presence of eptifibatide (mean ± SD,* n* = 3, *P < 0.05). (D) Platelet adhesion to von Willebrand factor (VWF) in the presence of eptifibatide (4 μm) under arterial flow conditions using fluorescently labeled human blood in the presence or absence of anti‐HSP47 (mean ± SD,* n* = 3, *P *> 0.05). (Ei, Eii) Platelets from HSP47^*flox/flox*^ and Pf4‐Cre; HSP47^*flox/flox*^ mice were stimulated by the addition of 1 μg mL ^−1^ CRP‐XL for 90 s. Immunoblot analysis was performed to detect total Syk phosphorylation using a phosphospecific Syk (Tyr 525/526) antibody. Data represent mean SYK 525/526 band intensity ± SD (*n* = 3). Student's *t*‐test, **P < 0.01. The band intensity of actin and Syk was used to control for protein loading levels.

To examine the role of HSP47 in platelet reactivity under physiologically relevant conditions in the presence of other plasma components and other blood cells, DiOC6‐labeled blood from mice lacking platelet HSP47 and control mice was perfused over collagen. Epifluorescence microscopy images were analyzed by calculating the sum intensity of fluorescence as a measure of thrombus formation. Figure 6(Ai) shows the representative fields of view. The sum intensity of fluorescence of thrombi formed with blood from Pf4‐Cre; HSP47^*flox/flox*^ mice was reduced by ~50% at 10 min (Fig. 6Aii) in comparison to HSP47^*flox/flox*^ mice. Similarly, DiOC6‐labeled whole human blood was perfused through collagen‐coated capillaries in the presence of HSP47 inhibitors. The volume of thrombi formed in SMIH‐treated blood was reduced by 32% compared with vehicle‐treated blood (Fig. 6Bi), and at a concentration of 5 μg mL^−1^ of anti‐HSP47 the mean thrombus volume was reduced by 27% compared with control IgG Fig. 6Bii).

### HSP47 supports platelet adhesion to collagen independently of GPIb

Platelet thrombus formation is defined by the deposition of platelets on collagen, with subsequent activation leading to recruitment of nearby platelets. Hence, it is the initial monolayer of platelets that may be most likely to influence the growth of the thrombus formation. Therefore, we investigated whether HSP47 plays a role in platelet adhesion to ascertain whether inhibition of HSP47 reduced the size of thrombi by preventing initial platelet–collagen interactions. *In vitro* thrombus formation assays were performed in the presence and absence of the integrin α_IIb_β_3_ inhibitor eptifibatide (4 μm). Platelet accumulation in the presence of eptifibatide (4 μm) prior to treatment with 20 μm SMIH or inhibitory antibody was reduced because of the lack of platelet–platelet interactions. Figure 6(Ci) shows representative fields of view from confocal microscopy. The levels of platelets adhered to collagen were reduced by 32% in the presence of 20 μm SMIH compared with vehicle‐treated blood (Fig. 6Cii) and by 54% in the presence of anti‐HSP47 (5 μg mL^−1^) compared with control IgG (Fig. 6Ciii). These data suggest that HSP47 influences the adhesion of platelets to collagen and thereby contributes to subsequent activation.

It is possible that HSP47 inhibitors prevented platelet–collagen interactions via the VWF receptor GPIb. To test this, eptifibatide‐treated (4 μm) whole blood was perfused over VWF‐coated capillaries in the presence of anti‐HSP47 or control IgG (Fig. 6D). Platelet adhesion to VWF, which was quantified from fluorescence intensity, was unaltered by HSP47 inhibition.

### HSP47 modulates GPVI signaling

To determine whether early signaling events following GPVI ligation were altered, the phosphorylation of SYK 525/526 was analyzed in platelets lacking HSP47. Control mouse platelets (HSP47^*flox/flox*^) stimulated with CRP‐XL (1 μg mL^−1^) showed an increase in tyrosine 525/526 phosphorylation at 90 s. However, HSP47‐deficient platelets (Pf4‐Cre; HSP47^*flox/flox*^) showed reduced phosphorylation of SYK 525/526, consistent with attenuated GPVI signalling (Fig. 6Ei, Eii). Furthermore, levels of PKC substrate phosphorylation on serine residues following CRP‐XL stimulation in Pf4‐Cre; HSP47^*flox/flox*^ platelets were reduced compared with HSP47^*flox/flox*^ (Figure S6Ai, Aii).

### Inhibition or deletion of platelet HSP47 reduces laser‐induced thrombosis

For intravital microscopy in mouse cremaster muscle arteriole experiments, laser injury was optimized to expose the extracellular matrix (ECM). Collagen type IV immunoreactivity was detectable within 10 s after laser injury (Figure S6B).

**Figure 7:**
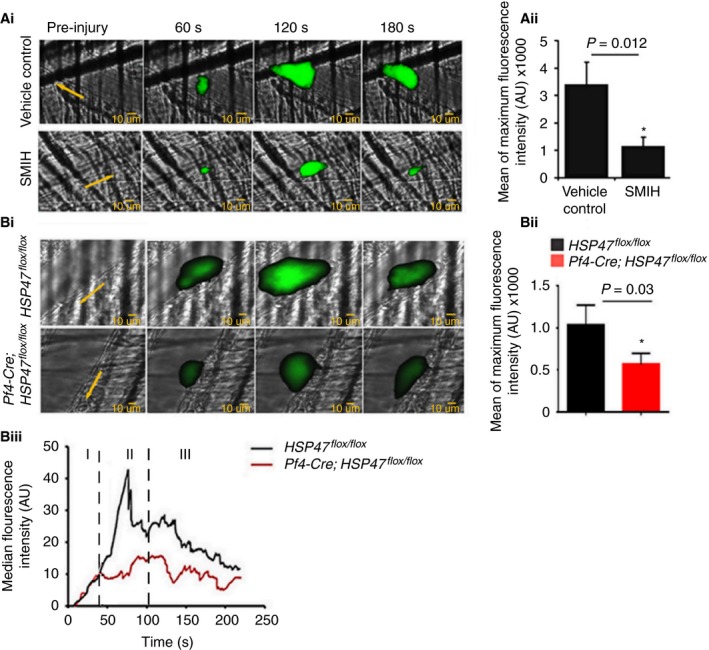
Absence or inhibition of HSP47 reduces thrombosis. (Ai) Thrombosis was measured following laser injury of cremaster muscle arterioles by intravital microscopy. C57BL/6 mice were administered small molecule inhibitor of HSP47 (SMIH) or vehicle control via cannula prior to injury. Platelets were labeled by infusion of Alexa Fluor 488‐conjugated anti‐GPIb antibody. Representative images of thrombi from vehicle control and SMIH (20 μm in blood volume) following injury are shown. (Aii) Data represent mean maximum fluorescence, *n* = 27 thrombi from each group, *P* = 0.012. (Bi) Intravital microscopy was performed on Pf4‐Cre; HSP47^*flox/flox*^ and HSP47^*flox/flox*^ mice. (Bii) Mean of maximum fluorescence intensity was measured from 12 thrombi from HSP47^*flox/flox*^ mice and 15 thrombi from Pf4‐Cre; HSP47^*flox/flox*^ mice (*n *= 3 mice) (Student's *t*‐test, *P* = 0.031). Images are ×60 magnification. (Biii) The kinetics of the platelet accumulation during thrombus growth was analyzed in HSP47^*flox/flox*^ and Pf4‐Cre; HSP47^*flox/flox*^ mice. Each curve represents the median integrated platelet fluorescence from 12 thrombi from HSP47^*flox/flox*^ mice (black) and 15 thrombi from Pf4‐Cre; HSP47^*flox/flox*^ mice (red) (from three separate mice). Fluorescence intensity of platelets in arbitrary units is presented as a function of time. [Color figure can be viewed at wileyonlinelibrary.com]

Anesthetized male C57BL/6 mice were injected intravenously with either SMIH or vehicle control prior to vessel wall injury. Time‐resolved images were captured using intensified intravital fluorescence microscopy. Mean of maximum fluorescence (maximum platelet recruitment) obtained from individual thrombi was reduced by 66% in the presence of SMIH compared with vehicle control in mice (Fig. 7Ai, Aii). Thrombosis upon laser injury was also assessed in mice lacking HSP47. Mean of maximum fluorescence (maximum platelet recruitment) obtained from individual thrombi was reduced by 54% in Pf4‐Cre; HSP47^*flox/flox*^ mice compared with HSP47^*flox/flox*^ mice (Fig. 7Bi, Bii, Biii). The initial kinetics (up to 45s) of thrombus formation (phase I in Fig. 7Bii) were unaltered in the absence of HSP47, consistent with no involvement of HSP47 in GPIb‐dependent platelet capture (Fig. 6D). The following growth phase of thrombus formation (phase II in Fig. 7Bii) was reduced. Beyond approximately 100 s the kinetics of thrombus decline were similar (phase III in Fig. 7Bii).

### Lack of HSP47 extends mouse bleeding times

**Figure 8:**
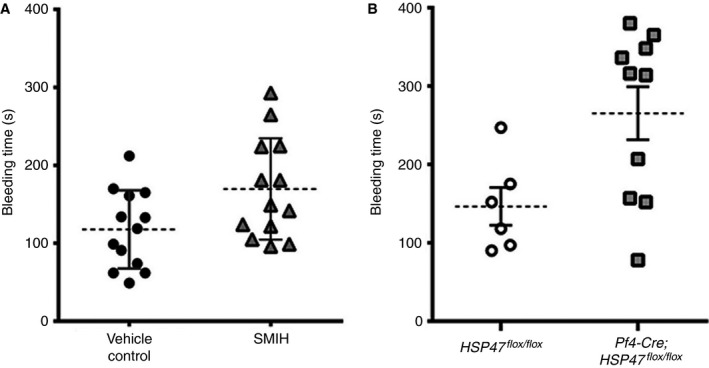
Inhibition of HSP47 extends bleeding times. Tail bleeding assays were performed on anesthetized C57BL/6 mice injected intravenously with small molecule inhibitor of HSP47 (SMIH) or vehicle control. (A) The graph represents bleeding time measured after mice were injected with SMIH (triangle) or vehicle control (circle) (*n* = 13 of each group, Mann–Whitney test, *P < 0.05). Tail bleeding assays were performed on anesthetized platelet HSP47‐deficient mice and control mice. (B) The graph represents bleeding time measured in HSP47^*flox/flox*^ (unshaded circle, *n* = 6) and Pf4‐cre; HSP47^*flox/flox*^ mice (square, *n* = 10) (Mann–Whitney test, *P < 0.05).

Because HSP47 plays an important role in thrombosis, disruption of platelet function may be expected to compromise hemostasis. Five minutes after intravenous injection of SMIH or vehicle to C57Bl/6 mice, tail biopsies were performed and bleeding time was recorded. Mean tail bleeding time increased by 31% (117 ± 13 s vs. 192 ± 30 s, Fig. 8A). Consistent with the inhibition of HSP47, platelet‐specific HSP47‐deficient mice had prolonged tail bleeding times by approximately 1.8‐fold in comparison with control mice (265.3 ± 106.9 s [platelet HSP47‐deficient mice] vs. 146.5 ± 58.99 s [control mice], Fig. 8B).

## Discussion

Platelet adhesion and activation on collagen involves platelet surface receptors, including GPIb‐V‐IX, GPVI and integrin α_2_β_1_, functioning in a coordinated and potentially synergistic manner 19, 20. Although HSP47 is primarily known for its function as a collagen binding protein, it also shares sequence homology with the serine protease inhibitor (serpin) family. However, sequence differences in the active site render the protein inactive as a serpin 21, as confirmed in protease inhibition studies with purified recombinant mouse HSP47 22. Using a range of *in vitro* and *in vivo* assays of platelet function, HSP47‐deficient mouse platelets and selective inhibitors, we provide compelling evidence for an important extracellular role for this protein, predominantly in collagen‐mediated platelet function.

In this study, platelet‐derived HSP47 was confirmed to bind to mature collagen type I fibrils, a collagen type present within the arterial wall 23, in accordance with the previous reports that HSP47 binds to collagen types I to V 24. A study by Nakai *et al*. 25 has shown the inability of HSP47 to interact with other constituents of the extracellular matrix such as laminin or fibronectin 25. Therefore, the actions of HSP47 in platelets are likely to be mediated through interactions with the collagenous component of the ECM.

Blockade of platelet HSP47 or its deletion in mice led to a reduction in platelet responses to collagen and CRP‐XL. Furthermore, *in vivo* studies using a laser injury model of cremaster muscle arterioles revealed an important role for HSP47 in thrombus formation. The initial phases of thrombus formation were found to be unaltered in the absence (or inhibition) of HSP47. The early phase of platelet entrapment at sites of injury is indirect and dependent on GPIb–VWF interactions. The interaction was found not to be regulated by HSP47, and therefore the initial phases of thrombus formation would be expected to be unchanged in the absence of HSP47 activity. The rapid thrombus propagation and growth that follows initial entrapment, a response that is dependent on direct interactions of collagens with GPVI and integrin α_2_β_1_, was reduced substantially in the absence of HSP47 activity. Hence, we propose that HSP47 strengthens and enhances platelet–collagen interactions following the initial GPIb‐dependent platelet entrapment.

It is currently unclear how HSP47 exerts this effect, whether through modulation of ligand structure, or indeed because it functions as an adhesion protein to enhance platelet–collagen interactions. Recent evidence indicates that GPVI can also bind to fibrin, hence we investigated whether HSP47 could alter platelet adhesion and spreading to fibrin 26. Deletion of HSP47 does not alter the platelet adhesion to fibrin (Figure S6Ci, Cii). Although the inhibition or deletion of HSP47 reduced thrombosis in mice, bleeding was also extended, indicating a fundamental role for this extracellular chaperone protein in thrombosis.

The escape of normally endoplasmic reticulum‐resident proteins to the extracellular environment is shared in platelets with thiol isomerases, a family of oxidoreductases that contribute to the folding of nascent proteins. These proteins are also implicated in the modulation of hemostasis and thrombosis 17, 27.The discovery that extracellular HSP47 modulates platelet responses selectively to collagen suggests that the ability of chaperone proteins to function in the extracellular environment may represent an hitherto unrecognized paradigm where such proteins may continue to ‘chaperone’ protein structure and function outside of the cellular secretory pathway. This may be important in a number of other (patho) physiological circumstances.

## Addendum

P. Sasikumar, W. J. Kaiser, L. M. Holbrook, A. Bye, K. S. AlOuda, A. J. Unsworth and T. Sage performed experiments. N. Kriek performed data analysis. R. Ushioda, K. Nagata and N. Kriek assisted in the development of the platelet HSP47 mouse model. P. Sasikumar, N. Kriek, R. W. Farndale and J. M. Gibbins designed the study and prepared the manuscript.

## Disclosure of Conflict of Interests

The authors state that they have no conflict of interest.

## Supporting information


**Fig. S1.** (Ai, Aii, Aiii, Aiv) Resting platelets express HSP47 on the cell surface.

**Fig. S2.** Anti‐HSP47 reduces platelet aggregation.

**Fig. S3.** Characterization of platelet‐specific HSP47‐deficient mice.

**Fig. S4.** Mouse platelets lacking HSP47 exhibited reduced platelet aggregation and fibrinogen binding in response to CRP‐XL.

**Fig. S5.** Confirmation of selectivity of HSP47 inhibitors.

**Fig. S6.** (Ai, Aii) HSP47 modulates platelet signaling in response to CRP‐XL activation.
