# Supplementary figures and images for: The chaperone protein HSP47: a platelet collagen binding protein that contributes to thrombosis and hemostasis
Source: J Thromb Haemost. 2018 Apr 15;16(5):946–59. doi: 10.1111/jth.13998 (PMC6434988; doi:10.1111/jth.13998)

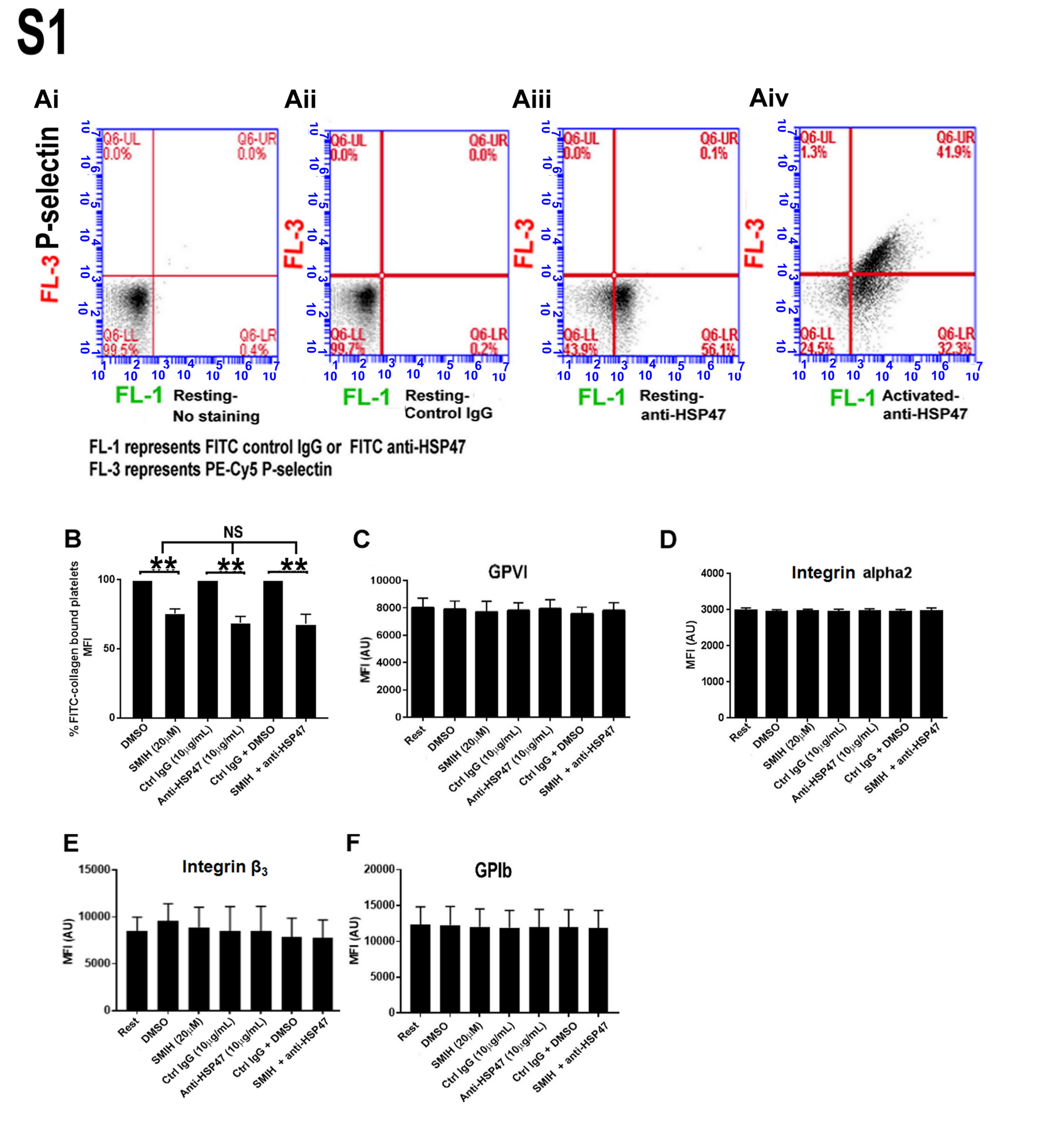

Supplement: Supplementary file 1 — Fig. S1. (Ai, Aii, Aiii, Aiv) Resting platelets express HSP47 on the cell surface. [file JTH-16-946-s001.tif]

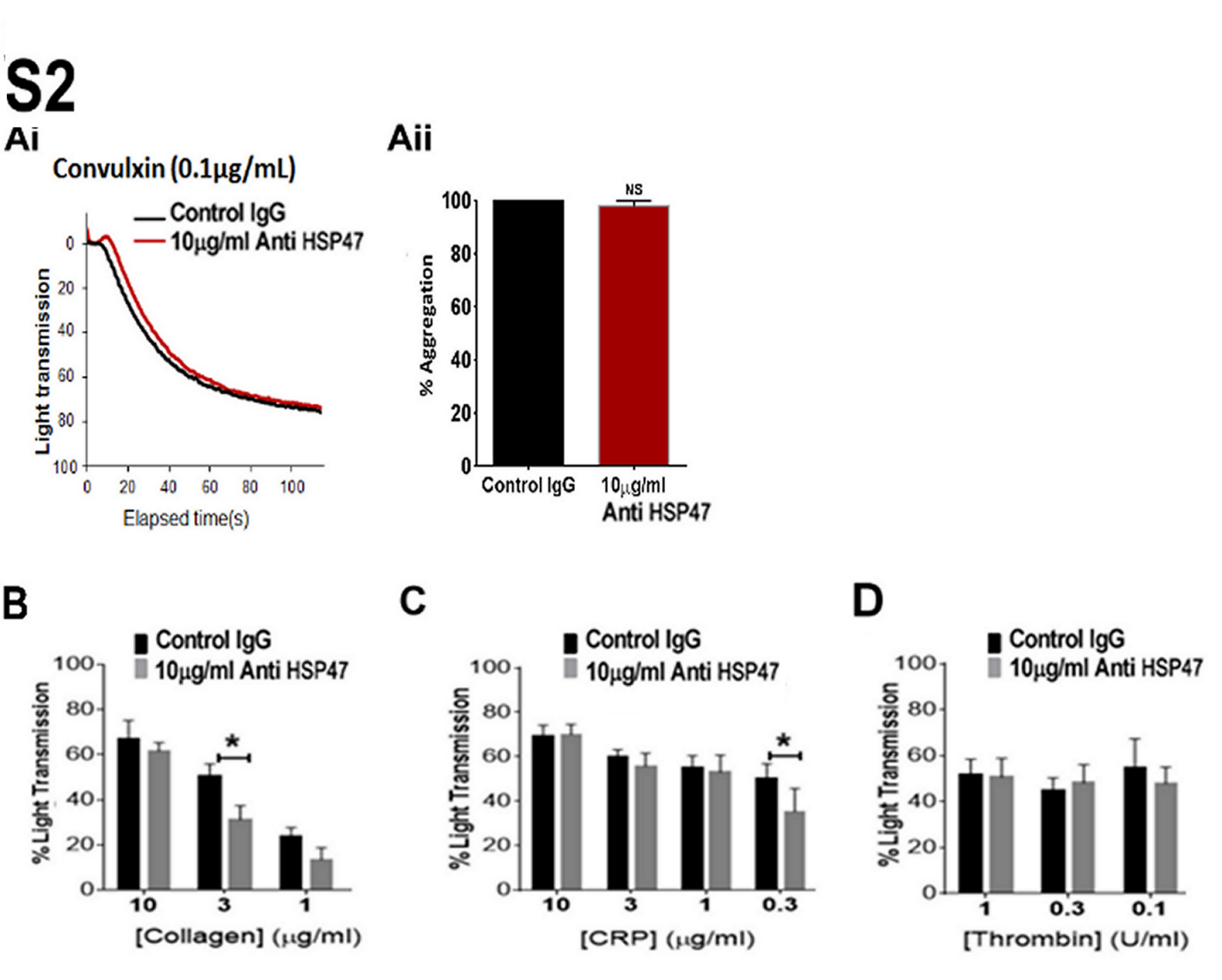

Supplement: Supplementary file 2 — Fig. S2. Anti‐HSP47 reduces platelet aggregation. [file JTH-16-946-s002.tif]

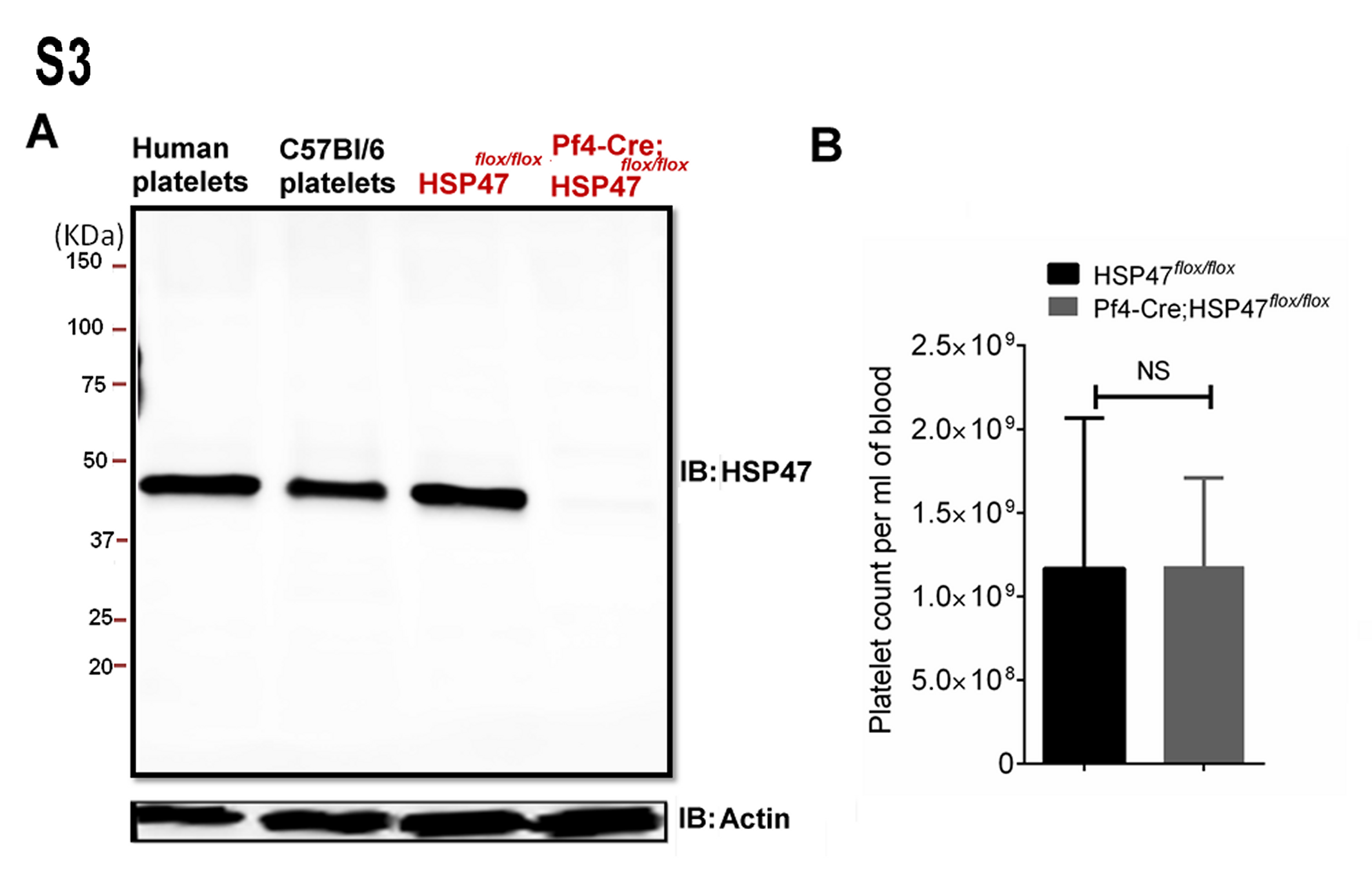

Supplement: Supplementary file 3 — Fig. S3. Characterization of platelet‐specific HSP47‐deficient mice. [file JTH-16-946-s003.tif]

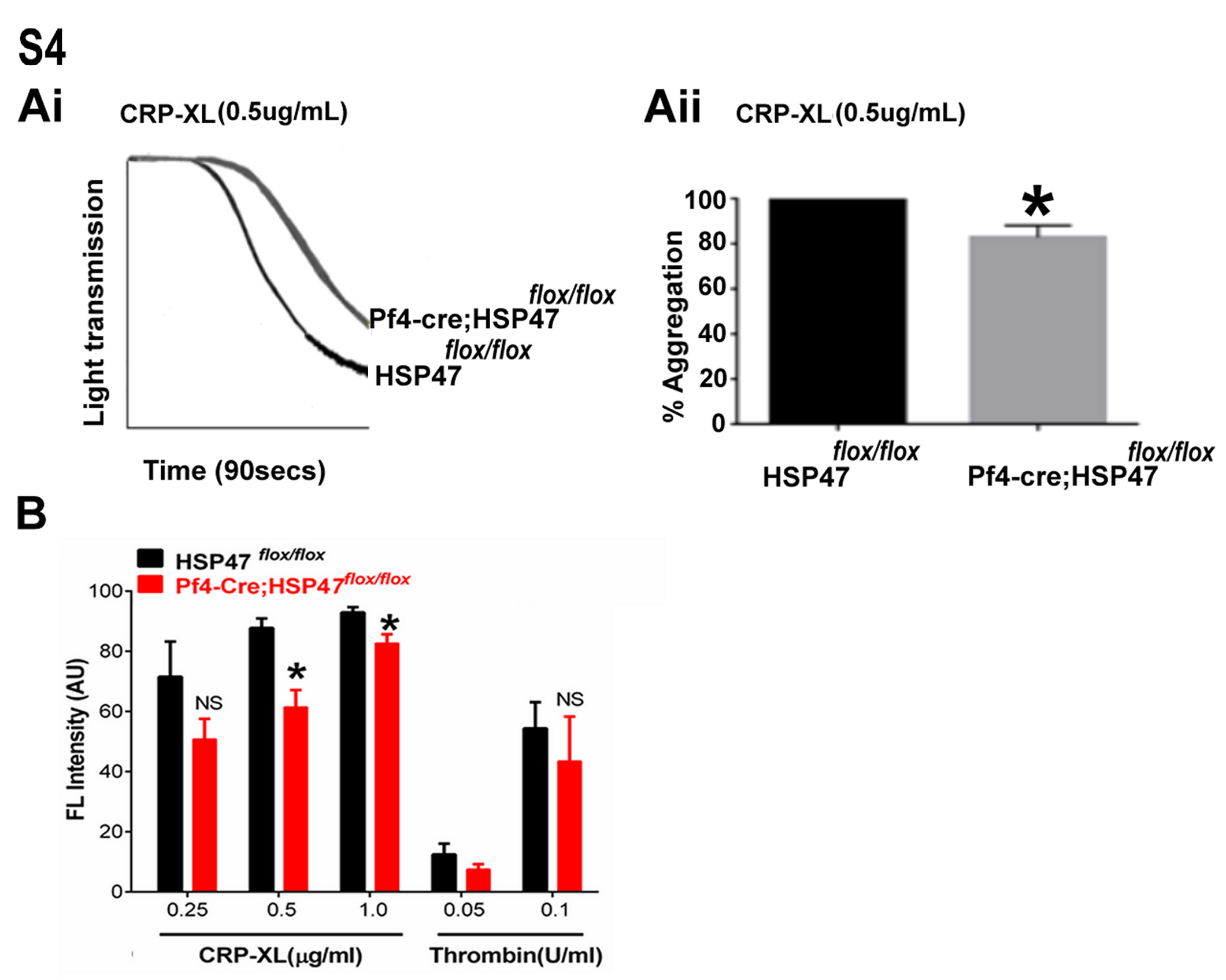

Supplement: Supplementary file 4 — Fig. S4. Mouse platelets lacking HSP47 exhibited reduced platelet aggregation and fibrinogen binding in response to CRP‐XL. [file JTH-16-946-s004.tif]

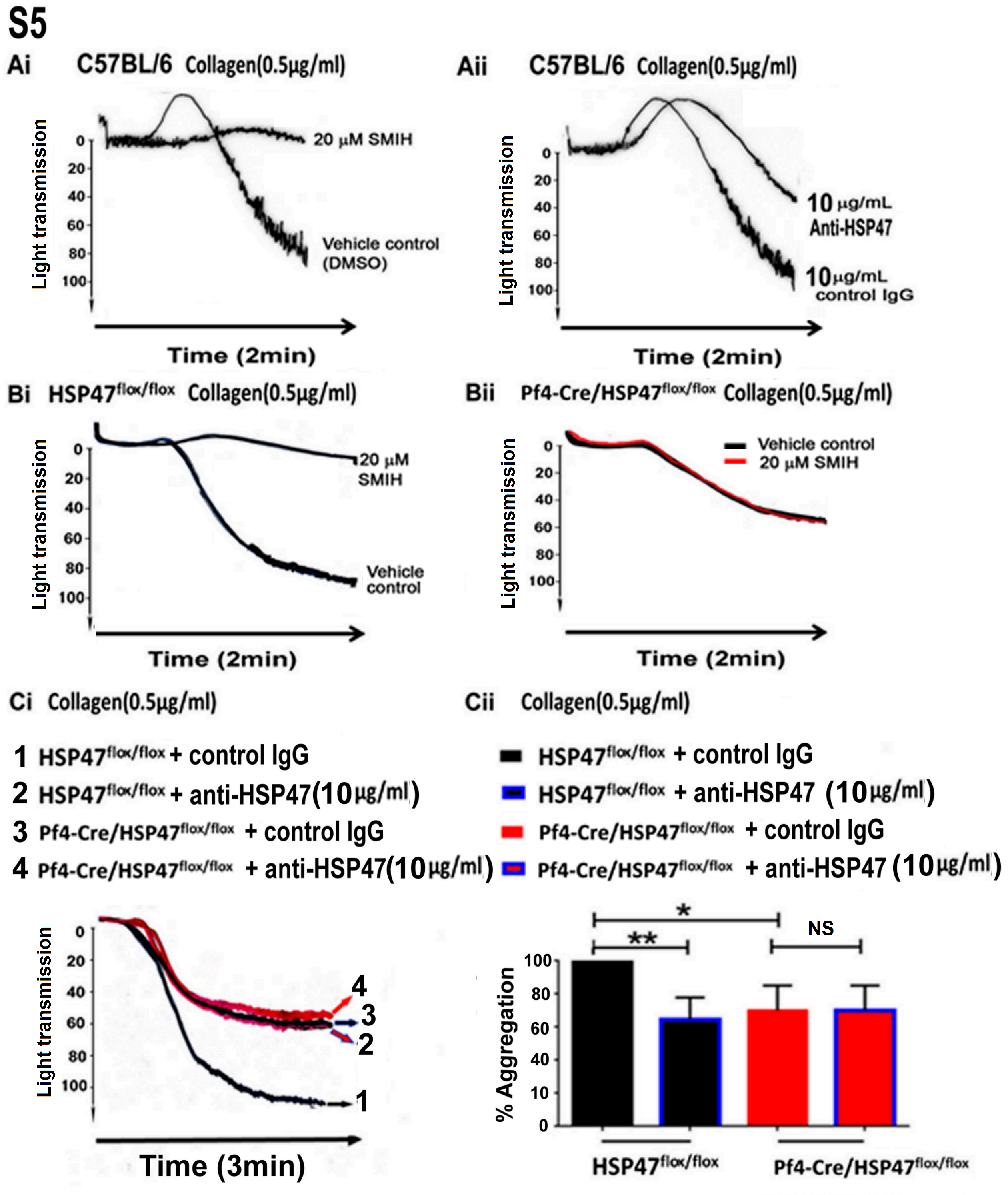

Supplement: Supplementary file 5 — Fig. S5. Confirmation of selectivity of HSP47 inhibitors. [file JTH-16-946-s005.tif]

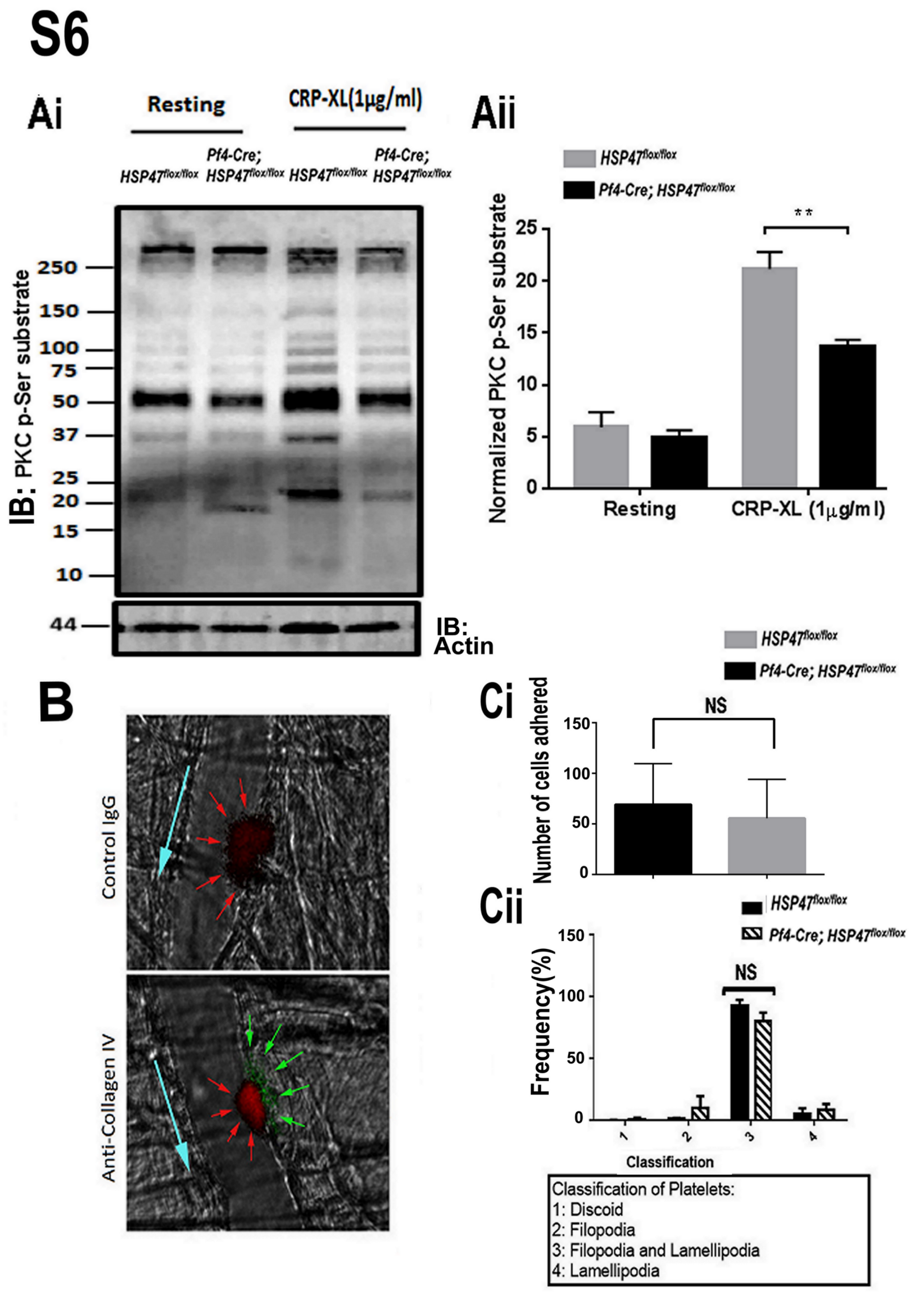

Supplement: Supplementary file 6 — Fig. S6. (Ai, Aii) HSP47 modulates platelet signaling in response to CRP‐XL activation. [file JTH-16-946-s006.tif]
